# Transcription factor-associated combinatorial epigenetic pattern reveals higher transcriptional activity of TCF7L2-regulated intragenic enhancers

**DOI:** 10.1186/s12864-017-3764-9

**Published:** 2017-05-12

**Authors:** Qi Liu, Russell Bonneville, Tianbao Li, Victor X. Jin

**Affiliations:** 1 Department of Molecular Medicine, University of Texas Health Science Center, 8403 Floyd Curl, San Antonio, TX 78229 USA; 2 College of Life Science, Jilin University, Changchun, 130012 China; 3 Biomedical Sciences Graduate Program, The Ohio State University, Columbus, OH 43210 USA

**Keywords:** T-cep, TCF7L2, Intragenic enhancer, PANC1, MCF7

## Abstract

**Background:**

Recent studies have suggested that combinations of multiple epigenetic modifications are essential for controlling gene expression. Despite numerous computational approaches have been developed to decipher the combinatorial epigenetic patterns or “epigenetic code”, none of them has explicitly addressed the relationship between a specific transcription factor (TF) and the patterns.

**Methods:**

Here, we developed a novel computational method, T-cep, for annotating chromatin states associated with a specific TF. T-cep is composed of three key consecutive modules: (i) Data preprocessing, (ii) HMM training, and (iii) Potential TF-states calling.

**Results:**

We evaluated T-cep on a TCF7L2-omics data. Unexpectedly, our method has uncovered a novel set of TCF7L2-regulated intragenic enhancers missed by other software tools, where the associated genes exert the highest gene expression. We further used siRNA knockdown, Co-transfection, RT-qPCR and Luciferase Reporter Assay not only to validate the accuracy and efficiency of prediction by T-cep, but also to confirm the functionality of TCF7L2-regulated enhancers in both MCF7 and PANC1 cells respectively.

**Conclusions:**

Our study for the first time at a genome-wide scale reveals the enhanced transcriptional activity of cell-type-specific TCF7L2 intragenic enhancers in regulating gene expression.

**Electronic supplementary material:**

The online version of this article (doi:10.1186/s12864-017-3764-9) contains supplementary material, which is available to authorized users.

## Background

Numerous public data resources, including the ENCODE and Epigenomics Roadmap, have generated thousands of genome-wide data sets, and provided us with substantial quantities of data to study transcriptional and epigenetic patterns in different cell types at a genome-wide scale [1, 2]. Many studies have shown that epigenetic modifications play a central role in regulating gene expression, and are involved in a diversity of biological processes in the human organism [3, 4]. However, the “epigenetic code”, referring to the transcription of genetic information encoded in DNA is in part regulated by DNA methylation and histone modifications, has not yet been fully elucidated. Substantial efforts in cracking this code and uncovering its biological functions have been made, utilizing both in vitro and in silico methods [5–7]. One innovative and practical way is to segment the epigenome into various chromatin states [8–10], where each state is decoded as a specific combinatorial pattern of multiple epigenetic modifications. Such states or combinatorial patterns can reflect a variety of sets of genes’ expression levels, which are essential to establish and maintain distinct functions in chromatin [11, 12]. For instance, novel classes of enhancers have been identified through genome-wide pattern analyses [13–15]. Therefore, identification of epigenetic regulatory combinations and networks is increasingly important for understanding genome functions, chromatin components and molecular mechanisms.

Computational approaches and models have been a major force to uncover these complex epigenetic patterns. These contain probabilistic methods, such as ChromaSig, an unbiased clustering and alignment approach that finds over-represented epigenetic signatures [16]. More advanced machine learning approaches have been applied in recent work, such as hidden Markov models (HMM) [10] and Bayesian network methods [17]. The HMM has proved to be a good model in training models with numerous inputs, and that generate thousands of outputs [18–20]. For example, ChromHMM utilized a multivariate HMM to learn chromatin states and to output emission probabilities for each mark in each state and then to infer the number of combinatorial marks in each of all states [21]. Segway [17] applied dynamic Bayesian networks (DBN) to segregate the genome with a higher segment resolution, and derive different chromatin states from chromatin marks [22]. Although these computational methods have successfully been used to annotate chromatin elements, many of them suffer from significant limitations. For instance, some supervised learning methods cannot find de novo information. Some unsupervised learning methods train on small genomic regions such as Segway [17] and HMMSeg [23], or output a single mark’s probability such as ChomHMM [21]. None of these algorithms explicitly compare targets of a specific TF with different histone modification marks to qualitatively assess the association of that TF with active, repressive and elongation regions. Most TFs have narrow binding patterns compared to most histone modification marks, but are major regulators of gene expression [24, 25]. Therefore, by ignoring TF binding in the beginning of algorithm design or training, a method may erroneously consider it as less meaningful due to the lower probability of TF than chromatin states, or not incorporate TF binding information at all. As such, it is critical to develop an algorithm that considers a specific TF together with many different epigenetic marks.

In order to address TF-dependent epigenetic regulatory events, we have developed a novel algorithm and software tool, T-cep (Transcription factor-associated combinatorial epigenetic patterns), which applies a univariate HMM to identify combinatorial epigenetic patterns associated with cell type-specific TF targets in different cell types. To evaluate T-cep, we applied it on TCF7L2-omics data, including ChIP-seq data of TCF7L2, Pol-II, active chromatin marks (H3K4me1,3 and H3K27ac), repressive chromatin marks (H3K27me3 and H3K9me3), a mark of transcriptional elongation (H3K36me3) as well as DNase-seq for open chromatin regions in five cancer cell types.

TCF7L2 (transcription factor 7-like 2), an important component of WNT pathway, has been implicated in several human diseases including carcinogenesis, type 2 diabetes and bipolar disorder [26–29]. The WNT pathway is often constitutively activated in human cancers, such as colon, liver, breast, and pancreatic cancer [30], with high upregulation of TCF7L2. Several studies have shown tissue-specific alternative splicing of TCF7L2, suggesting that TCF7L2 may have different functional properties in different cells [31, 32]. In our previous studies, we have mapped genome-wide binding of TCF7L2 in six cell lines [33, 34]. However, the combinatorial epigenetic profile of TCF7L2 in these cancer types has not been well studied. One hypothesis is that TCF7L2 regulates its downstream target genes in a cell type-specific manner, i.e., utilizing different combinatorial patterns with various epigenetic environment cues in each cell type. Therefore, one way to test this hypothesis is to identify TCF7L2-assoicated combinatorial epigenetic patterns in a diverse set of cell types.

In this paper, we first describe the workflow of T-cep, then present training results on TCF7L2-omics data in five cancer cell types, and compare it with ChromHMM [21]. Finally, we perform functional validations using siRNA, RT-qPCR, co-transfections and luciferase reporter assays on selected gene loci. To the best of our knowledge, this is the first genome-wide combinational epigenetic pattern discovery study for cell type-specific TCF7L2 regulation.

## Results

### The workflow of T-cep

**Fig. 1:**
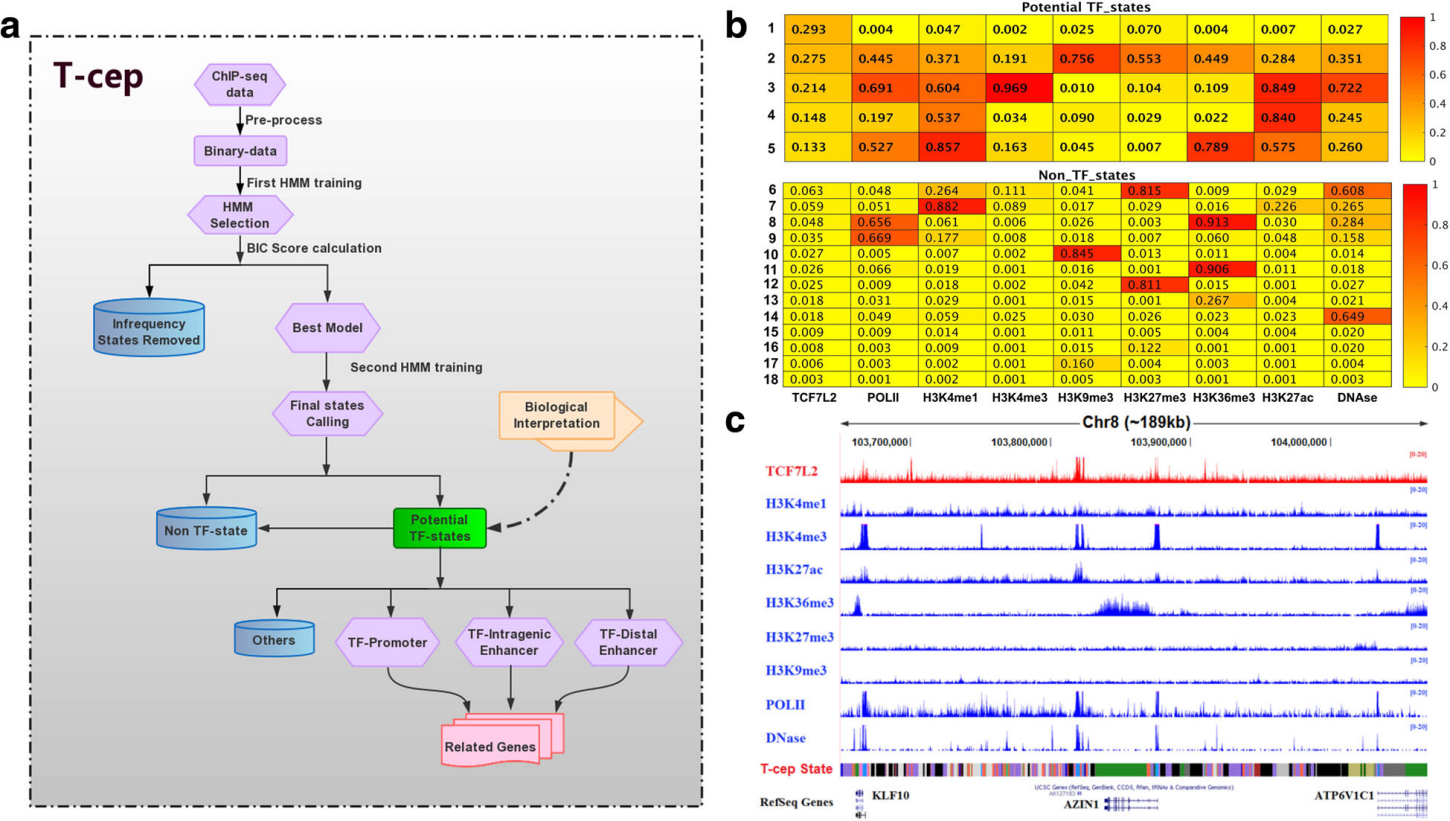
T-cep algorithm and training results for TCF7L2-omics data. **a** The workflow of T-cep algorithm. Shown is a schematic summary of the four steps needed for *de novo* identifying transcription factor associated chromatin state (TF-state). The approach begins with preprocessing the ChIP-seq data into an alphabet of 2^n^ observations, builds a HMM to generate a best selected model without infrequent state, re-estimate transition and emission probabilities and then annotate biological meaningful TF-states. **b** Result of T-cep approach. The emission probabilities of each mark are independent of others for the selected 18-state HMM. The mark probability of greater than 0.1 is considered to be associated with a chromatin state. **c** A screenshot of a genomic region in Chr8 of MCF-7 annotated by T-cep in UCSC genome browser. ChIP-seq of TCF7L2 and histone marks are in the first nine lines, while the tenth track represents the main output of the T-cep, annotated different states represented by different colors. RefSeq gene (HG19) genomic positions are shown in the last line

To identify TF regulated chromatin states through a variety of epigenetic marks, we have developed a novel computational method, T-cep, for annotating chromatin states associated with a specific TF. T-cep is composed of three key consecutive modules: (i) Data pre-processing, (ii) HMM training, and (iii) Potential TF-states calling (Fig. 1a). The detail of the algorithm is described in Methods.

#### Step 1: Data pre-processing

T-cep uses a univariate HMM, where each bin can only emit one probability value, which corresponds to a combination of different marks. An alphabet of 2^n^ (*n* = the number of epigenetic marks) observation symbols is constructed by enumerating each possible combination of marks (including no marks). We combine all ChIP-seq data in multiple cells as observations and translate them to such an alphabet of 2^n^ observations as an input for the next step. If necessary for computational efficiency, this alphabet can be simplified by removing symbols that correspond to mark combinations not present in the input data.

#### Step 2: HMM training

We initially train multiple HMMs for 300 iterations with T-cep, and select the best model based on the lowest BIC score. If desired, this model may be simplified by removing infrequent states. This is performed by eliminating the states which are called in very few bins (lower than 5% of the average number) and uniformly redistributing their transition probabilities to other states. We then re-train this derived HMM for another 100 iterations to produce the final model.

#### Step 3: Potential TF-states calling

The Viterbi decoding algorithm is used to output called states for all bins in the genome. The probabilities of each mark are futher calculated by marginalization for each of different cell types respectively. We chose states with a cutoff of probability greater than 0.1 for any individual mark for further investigation, as these are most likely to yield meaningful biological insights. Finally, the HMM states can be classified as potential TF-associated chromatin states (TF-states) and potential non-TF-associated chromatin states (non-TF-States).

### Identification of TCF7L2-associated chromatin states by T-cep

To demonstrate the accuracy of T-cep and evaluate its performance, we chose TCF7L2-omics data as a study case. TCF7L2 is an important component of the WNT pathway, and has previously been studied by our laboratory. Additionally, 45 datasets of omics-seq data are available for the training (Additional file 1: Table S1-S2, all are available in the ENCODE Consortium), including ChIP-seq of TCF7L2, six histone marks (H3K4me1, H3K4me3, H3K9me3, H3K27ac, H3K27me3, H3K36me3), Pol-II and DNase-seq in five cancer cell types, HCT116, HeLa, HepG2, MCF7 and PANC1, with a total of ~2.02 billion reads. For each of these cell types, we also obtained RNA-seq data from our previous study or publicly available sources [35, 36].

We initially trained five 25-state HMMs over the data with bin sizes of 750 bp for 300 iterations, and selected the best HMM with the lowest BIC score of 4.3247 • 10^7^ (Additional file 1: Table S3). The states of every bin in each genome were called after the first HMM training. Seven infrequent states (lower than 5% of the average) were then eliminated (Additional file 1: Table S4) to produce an 18-state HMM model with BIC 4.40718 * 10^7^. We then performed a secondary HMM training for 100 iterations, and the log-likelihood was calculated at each iteration to verify model convergence (Additional file 1: Figure S1). This produced the final model, with a BIC score of 4.31487 *10^7^. The transition probabilities and the emission probabilities of each final state are shown in Additional file 1: Figures S2-S3. The Pearson correlation between the HMM emission probabilities and the actual observation frequencies under each state was *R* ^2^ ≥ 0.97 for all cell types, except HCT116 with *R* ^2^ = 0.9545 (Additional file 1: Table S5 and Figure S4). We then divided emission probabilities for each mark independently by marginalization among combinations of marks probabilities and potential TF-states (Fig. 1b and Additional file 1: Figure S5). A comparison of a genomic region for the states annotated by T-cep and the actual ChIP-seq signals (Fig. 1c) clearly demonstrated the ability of T-cep to accurately capture epigenetic elements.

**Fig. 2:**
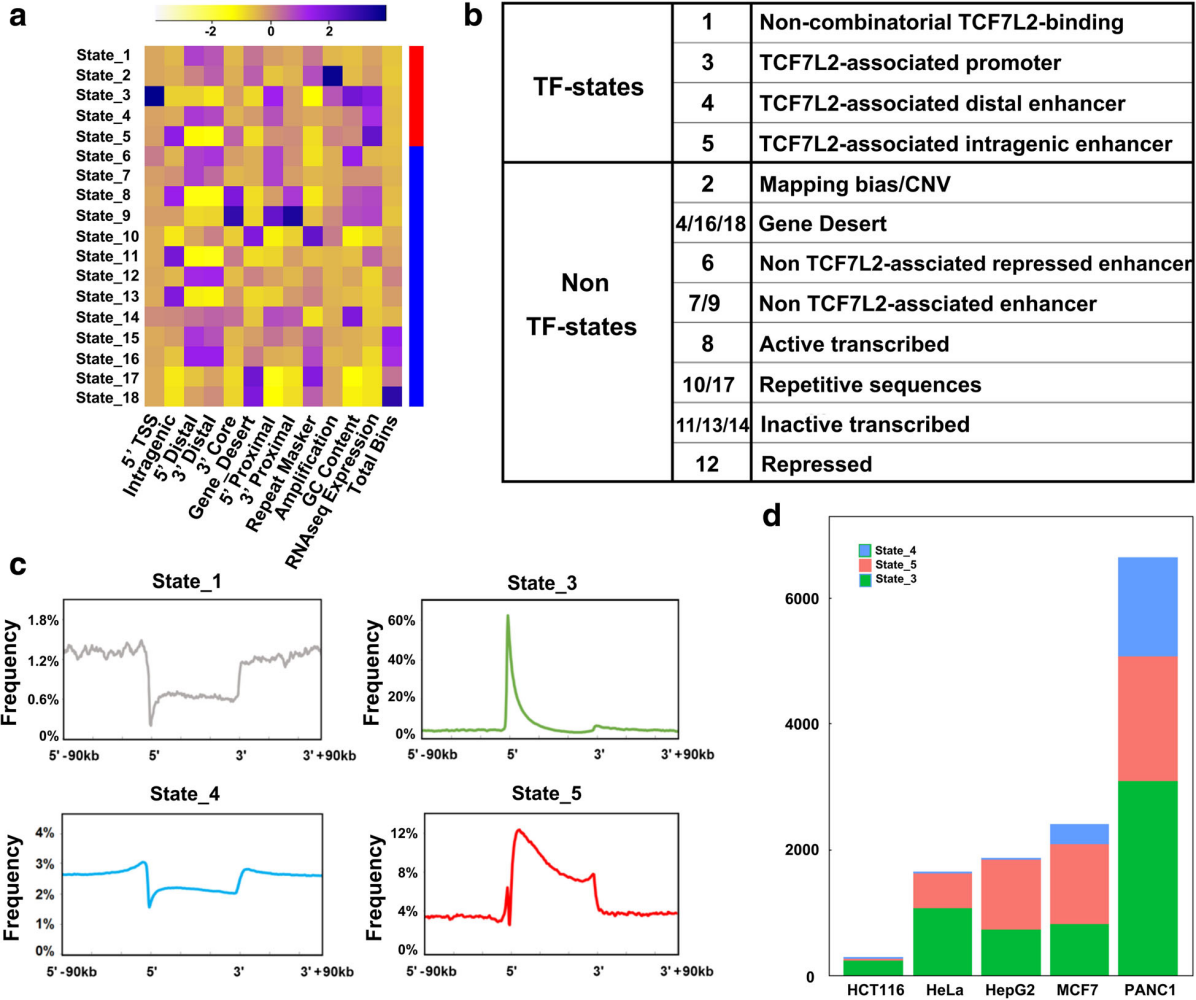
The annotation of chromatin states for TCF7L2-omics data. **a** The correlation with other genomic features, the distribution of each state bins as well as the average of gene expression for each of 18 states. The average gene expression is normalized by z-score and % total bin shows the percentage of each state in the human genome. **b** The biological interpretations of the HMM states and identification of four TCF7L2-assoicated States according to the emission probability and other genomic information. **c** The distribution of four TF-associated States on the gene structure. All genes’ length is normalized, representing the 5’–3’ region as gene body region and extending 90 kb of up/down steam for gene surrounding distribution. **d** Summary of bin distribution of three TCF7L2-associated states in five cancer cell types

We further examined the genomic characterization of potential TF-states using the annotated genomic regions defined in Additional file 1: Table S6, as well as the expression levels of their associated genes (Fig. 2a and Additional file 1: Figure S6). We were able to classify four states, 1, 3, 4 and 5, as TCF7L2-associated states, and the others as non-TCF7L2-associated states (Fig. 2b). State 3 was assigned as TCF7L2-associated promoter because of its higher frequency in 5’TSS regions (44.2% relative to other states) and its high emission probabilities for Pol-II and H3K4me3 (Fig. 2a). State 1 was classified as TCF7L2 binding, non-combinatorial TF-state, as its emission probability is only high for TCF7L2. We classified states 4 and 5 as TCF7L2-associated enhancer states due to their higher emission probabilities for the enhancer marks H3K27ac and H3K4me1. Location distribution showed most bins (75.9%) of state 5 were in gene body regions while the associated genes have a higher average gene expression as well as its high emission probabilities for enhancer marks H3K4me1, H3K27ac and gene body mark, H3K36me3 (Fig. 1b). Thus, state 5 was annotated as TCF7L2 intragenic enhancer. State 4 was classified as a TCF7L2 distal enhancer since 67.9% bins of state 14 were outside intragenic regions. Except those bins in Gene Desert regions, there were clearly more bins in 5’Distal regions than in 5’Proximal and 3’Distal regions. State 2 was classified as mapping bias/CNV due to its high emission probabilities for all active and repressive marks, where marks such as Pol-II and H3K9me3 were not expected to co-occur, as well as due to the relatively high proportion of its bins in known amplified regions.

We also identified some non-TCF7L2-associated states (non-TF-states). States 7 and 9 were classified as non-TCF7L2-associated enhancers. State 9 with a high Pol-II was assigned as an active non-TCF7L2-associated enhancer, while state 7 was likely to be an inactive non-TCF7L2-associated enhancer. Interestingly, the average gene expression levels of both states are less than those of the TCF7L2-associated states. State 6 was classified as a non-TCF7L2-associated bivalent state or poised enhancer due to its high emission probability of both repressive mark H3K27me3 and enhancer mark H3K4me1, and its lower average gene expression. All the other non-TCF7L2-associated states were assigned for their possible functions as well (Fig. 2b).

The annotation of chromatin states is further supported by the location analysis of bins within each state relative to genes (Fig. 2c). All genes’ length is normalized by Virtual bin Creation and the 5’-3’ region represents the whole gene body region and the distal region is extended to each to 90 kb upstream of 5’TSS or downstream of 3’TSS. Clearly, it is showed more than 60% of state 3 bins were located at or near the 5’TSS of each gene, (the green line), in which we interpreted it as promoter. State 5 is represented with the red line, in which its bins were distributed mainly in gene body region, thus called as intragenic enhancer. State 4 as distal enhancer shown in the blue line was mainly located in distal regions, and state 1 was infrequently in any of gene body or intragenic regions (Fig. 2c and Additional file 1: Figure S7).

In the other aspect, we wanted to re-examine whether the distribution in proportion of numbers of three annotated functional states is actually in their expected genomic regions. Indeed, State 3 bins are only in 5’TSS regions (131,086 bins), state 5 bins in intragenic regions (206,644 bins) and state 4 bins in 5’Distal regions (70,812 bins) for further analysis. Moreover, we observed in each cell type the distribution of these three TF-States in order 3/5/4 is the following: 239/32/26 for HCT116, 1061/559/30 for HepG2, 737/1109/29 for HeLa, 802/1265/327 for MCF7 and 3093/1972/1581 for PANC1 (Fig. 2d). Our results indicate the distribution in proportion of numbers of each of three functional states may be cell type specific.

### Gene enrichment and pathway analyses of TCF7L2-associated chromatin states

**Fig. 3:**
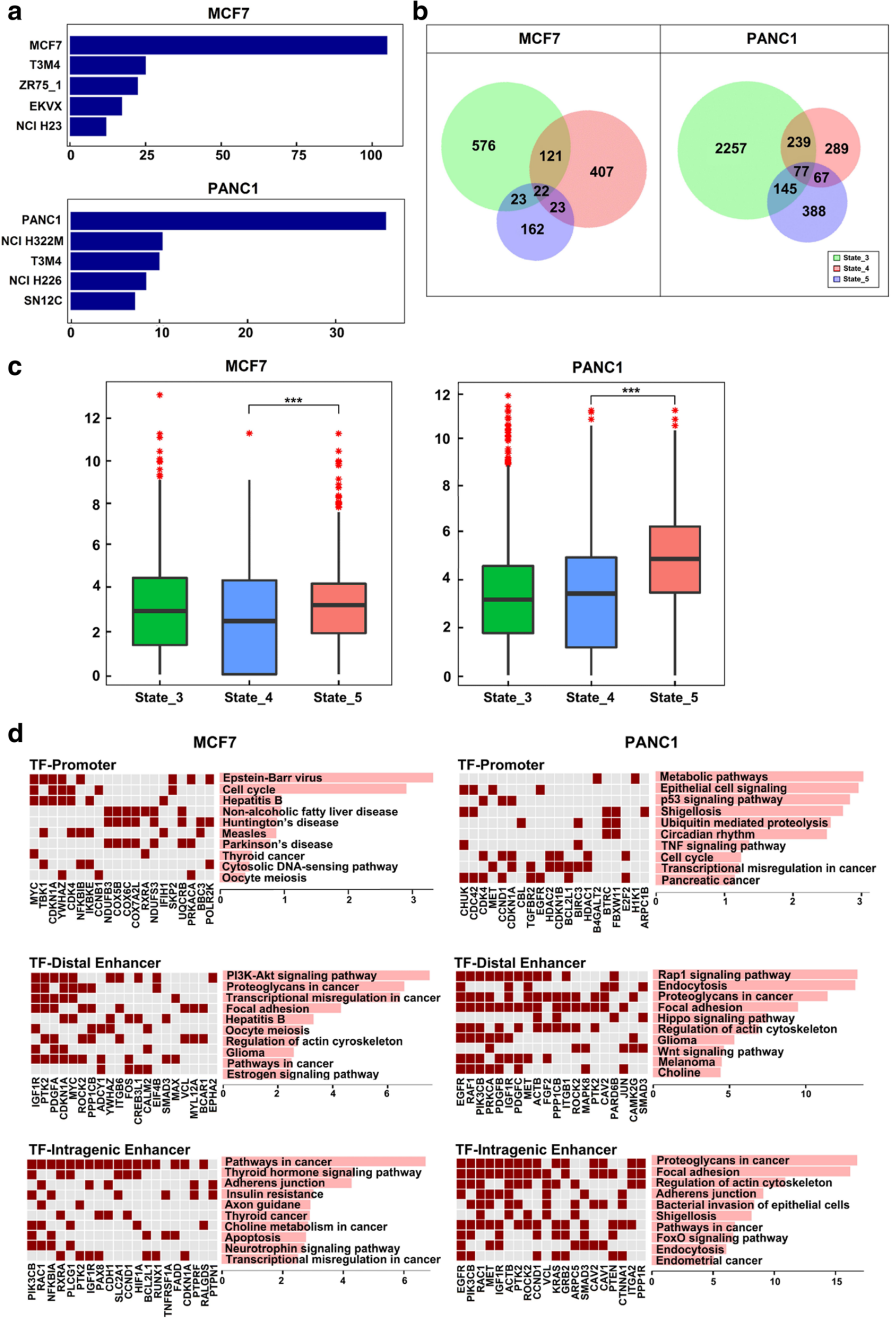
Genes with TCF7L2-associated states in MCF7 and PANC1 cells. **a** Identification of gene associated with TCF7L2-states in NCI-60 cell showing cancer type specificity. **b** A Venn diagram showing an overlapping set of genes among TCFL2-associated States in two cancer cell lines. **c** Boxplot of gene expression level showing TCF7L2-associated intragenic enhancers with the highest expression among three TCF7L2-associated states. Expression levels were log2-transformed, with genes having expression level <1 considered to have an expression level of 1. **d** The KEGG pathways for TCF7L2 promoters, TCF7L2 distal enhancers and TCF7L2 intragenic enhancers

Next, we wanted to examine in silico the biological functions of TCF7L2-associated chromatin states. We chose MCF7 and PANC1 cell types for further analysis as they had the highest number of genes associated with states 3, 4, and 5, where the number of genes associated with states 3/4/5 is 742/230/573 for MCF7 and 2718/677/672 for PANC1 respectively (see the list in Additional file 2). Firstly, we checked the cell type specificity of all of the genes. Interestingly, when examined in the National Cancer Institute’s NCI-60 cell lines [37], MCF7 cell line was the top category for genes selected in MCF7 cells, and PANC1 cell line was the top category for its selected genes (Fig. 3a). A Venn diagram of examining these same genes within each cell line showed a little among genes associated with each state, with only 22 genes in MCF7 and 77 in PANC1, respectively, associated with all three states (Fig. 3b). We also found that the genes associated with these three different TCF7L2 states in MCF7 and PANC1 cells are different, suggesting cell type specificity of TCF7L2 activity. Moreover, when we examined the average expression levels of all genes associated with each state, we found that the overall gene expression level of state 5 genes was the highest in all selected cell types (p-value < 0.0001) and much higher than genes associated with state 4 or state 3. Our result suggested that TCF7L2-regulated intragenic enhancers may play a prominent role in upregulating gene expression than TCF7L2-regulated distal enhancers (Fig. 3c).

We further examined the biological function of the TCF7L2-regulated genes by KEGG pathway enrichment analysis [38, 39]. Our results demonstrated that the enriched pathways are different between these three categories with cell type specificity (Fig. 3d). In overall, pathway enrichments in MCF7 cells were more involved in cancer initiation and cell cycle pathways, while those in PANC1 cells were more related to the metabolic pathway and cell adhesion. Interestingly, TCF7L2-regulated promoters are more enriched with well-known specific cancer initial development such as Epstein-Barr virus infection in breast cancer and metabolic in pancreatic cancer [20, 40, 41]. However, TCF7L2-regulated intragenic enhancers included advanced stage markers in cancers such as Thyroid hormone signaling and Proteoglycan in cell adhesion [42, 43], whereas TCF7L2-regulated distal enhancers are enriched with the general signaling in cancer development such as PI3K-AKT pathway and Rap signaling [44]. These results suggested that genes associated with TCF7L2 regulated intragenic enhancers may be more relevant to metastasis and cancer progression dependent on a specific cancer type.

### Comparison with ChromHMM

To compare T-cep with other published software tools, we chose ChromHMM [45] since it is a widely used software tool, is powerful in genomic segmentation with a user-friendly application, and can take different types of epigenetic marks (histone modification, transcription factor, DNA methylation and et al) as combinatorial inputs. However, when coming to the question of segmentation involved in a specific TF, the method has some limitations. 1) Given that the number of binding sites for most TFs are much less than those of histone modification sites at a whole genome, thus treating TF same as histone marks may lose the specific TF characteristics. 2) In ChromHMM, the independence between marks is transformed into a combination of probabilities in a given state. This would indicate that, for example, those regions showing low probability of TF binding are combined with the regions without TF binding because both have similar probabilities of other marks. Such output is contradicted to the actual combination. 3) Since there is no specific rule for determining the final threshold of segmentation, this may result in various outputs for the same data samples. In contrast, T-cep is designed to consider all possible events without ignoring any low number of marks. In addition, TF-state is not combined with other similar combinations since T-cep focuses on TF associated combinatorial states.

We trained our TCF7L2-omics data on ChromHMM with both 25-state model and 18-state HMM. We found that ChromHMM output similar patterns for two models, indicating the 18-state model is optimized for segmentation (Additional file 1: Figure S9). We matched the output of 18 states for both tools in order to show a direct side-to-side comparison. We also adopt the following metrics for the evaluation in order to show the TF-specific segmentation: 1) whether the tool is able to identify potential TF-states; 2) how many TF-state bins are able to be recovered in the annotated functional state; and 3) what is the quantitative correlation between annotated functional TF-state and gene expression level.

**Fig. 4:**
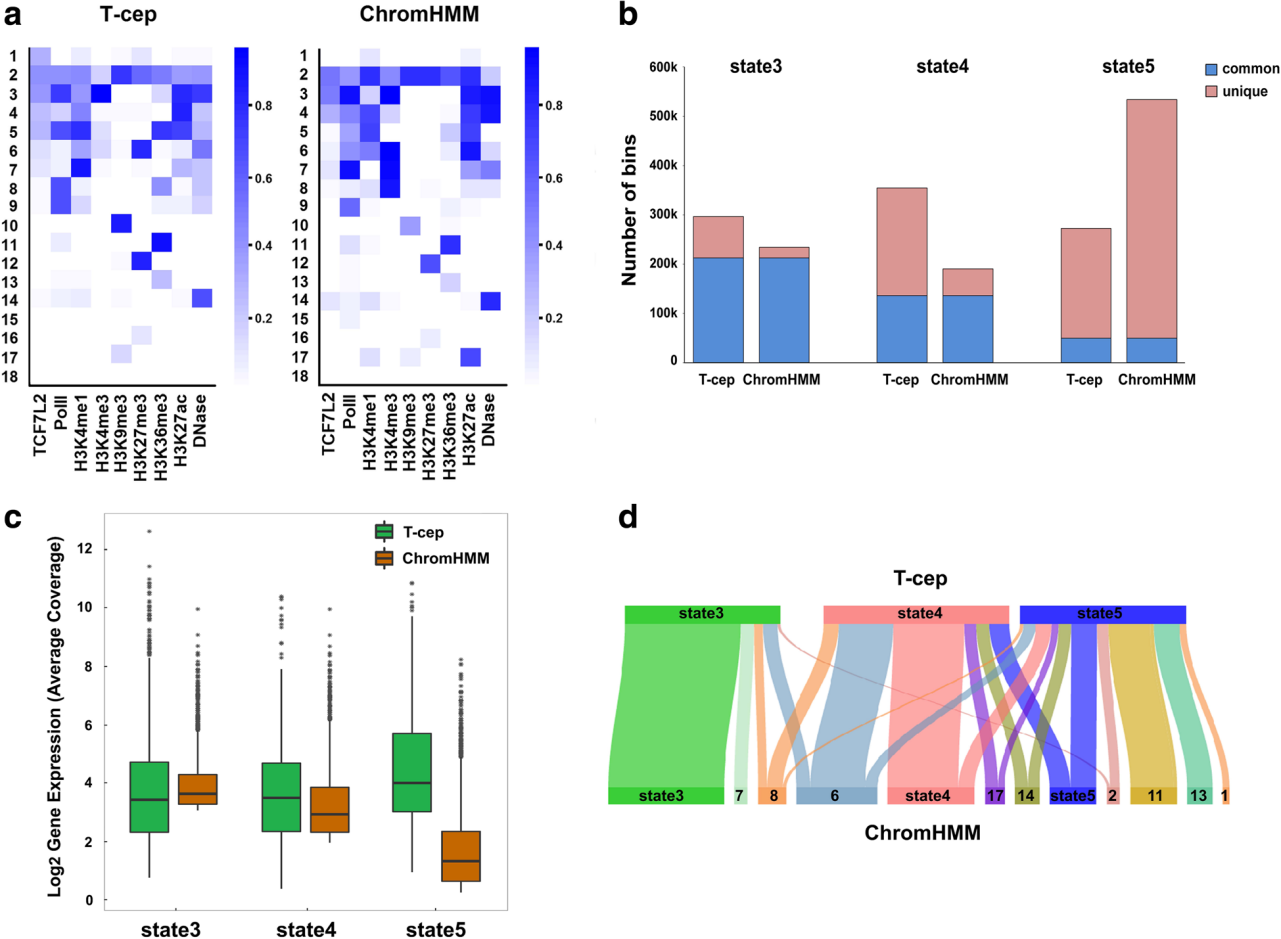
A comparison of T-cep with ChromHMM. **a** The emission probabilities of 18 states trained by T-cep and ChromHMM respectively. **b** The common and unique number of TF-state bins in each of three TCF7L2-associated functional states between T-cep and ChromHMM. **c** Boxplot of gene expression associated with those unique bins in each of three TF-states for T-cep and ChromHMM respectively. **d** Three TF-states’ segmentation (regions) identified by T-cep corresponding to states identified by ChromHMM in PANC1 cells

Despite that both methods can identify TCF7L2-associated states. T-cep were able to annotate four TF-states, states 1, 3, 4 and 5 and ChromHMM only annotated two TF-states, states 3 and 4 (Fig. 4a). Both methods identified a TCF7L2-associated promoter state (state 3) with a high probability of H3K4me3, and a TCF7L2-associated distal enhancer state (state 4) with highly enriched H3K27ac and H3K4me1. Although ChromHMM assigned state 5 as an intragenic enhancer with a high probability of H3K27ac, H3K4me1 and H3K36me3, the intragenic enhancer state is not correlated well with TCF7L2 binding. This clearly showed that ChromHMM missed an intragenic TF-enhancer state for TCF7L2-omics data. We also observed that a non-combinatorial TCF7L2-assoaited state, state 1, identified by T-cep was missed by ChromHMM. We then examed the number of bins recovered within each of annotated functional states and found T-cep had more bins for state 3 (296,248 vs 234,750 bins) and state 4 (354,573 vs 190,683 bins) respectively than ChomHMM did, while ChromHMM got more bins for state 5 (534,683 vs 272,409 bins) (Fig. 4b). For promoter regions related to state 3, we observed that a majority of the bins were common between T-cep (71.9% of all state 3 bins) and ChromHMM (90.8% of all state 3 bins), and only 83,175 and 21,677 bins were unique annotate in each of two methods respectively. For intragenic enhancer regions related to state 5, these two methods shared much less common bins with only 49,382 bins (18.1% for T-cep and 9.2% for ChromHMM) despite there are more bins overall in state 5 (Fig. 4b). The gene expression associated with those unique bins showed that the genes associated with promoters have a slightly higher for ChromHMM while those associated distal and intragenic enhancers are much higher for T-cep. Interestingly, genes associated with TCF7L2-regulated intragenic enhancers (state 5 in T-cep) showed the highest expression, indicating only T-cep is capable of identifying a specific set of TCF7L2-regulated intragenic enhancers (Fig. 4c). Further, we compared the overlapping and distinction of PANC1 cell segmentation between two methods. Clearly, promoter segmentation is similar between two methods, but enhancer segmentations are dramatically different. We found that state 4 in T-cep was mainly aligned to state 4 and state 6 in ChromHMM while state 5 in T-cep is split into several states in ChromHMM (Fig. 4d). These comparisons clearly showed the advantage of T-cep that can identify specific TF-associated states due to its consideration of a TF within the algorithmic design. Our results also pointed out a major difference between two methods such that ChromHMM is focused on annotating more functional combination of marks, while T-cep considers more influences of a specific TF on functional chromatin states.

### Functional validations of TCF7L2-associated intragenic enhancers

**Fig. 5:**
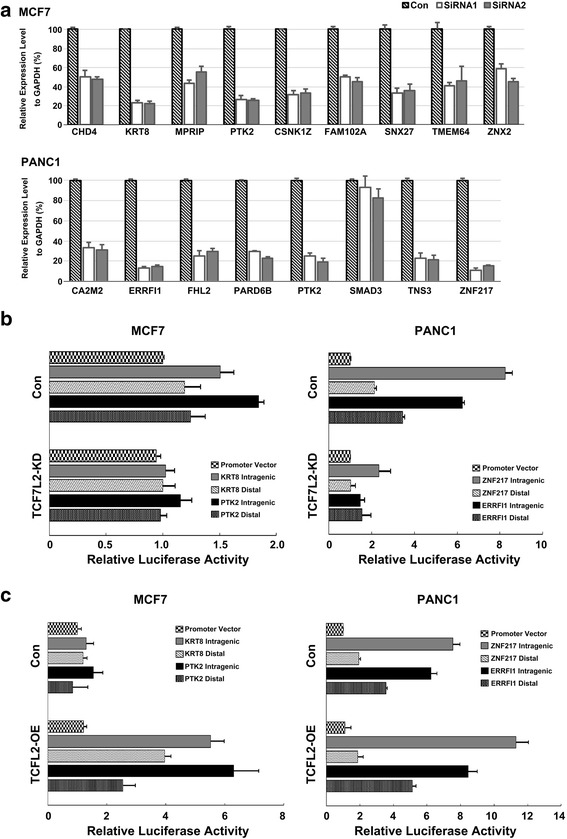
Functional validations of the predicted TCF7L2 enhancers by T-cep. **a** Quantitative PCR analysis of selected gene associated with TCF7L2-enhancers in MCF7 and PANC1 cells after TCF7L2 knockdown. Selected genes contain both of TCF7L2 intragenic and distal enhancers for further analysis. All data are normalized by GAPDH mRNA and represent the average of three independent experiments with p-values less than 0.05 (*t*-test). **b** Luciferase reporter activity assay in TCF7L2 knockdown cells. MCF7 and PANC1 cells are transient transfected with intragenic or distal TFE-pGL3 vector at the TCF7L2-siRNA or non-targeting siRNA conditions. **c** Luciferase reporter activity assay in TCF7L2 overexpression cells. Intragenic or distal TF-enhancer-pGL3 vector and TCF7L2-pcDNA or empty vector are transient transfected into MCF7 and PANC1 cells

Using our T-cep method, we were able to identify a set of TCF7L2-associated distal and intragenic enhancers in MCF7 and PANC1 cells respectively. In order to compare the activity between intragenic and distal enhancer, we chose the genes having both intragenic and distal enhancer regions, nine in MCF7 and eight in PANC1 cells, respectively (Additional file 1: Table S7). We first performed the knockdown TCF7L2 experiment using small interfering RNA (siRNA) in these two cell lines and then used quantitative PCR assays to confirm these genes are actually regulated by TCF7L2 (Additional file 1: Figure S8). Our results showed that 94.1% (16/17 genes except SMAD3) of gene expressional levels were significantly decreased (*p* < 0.0001) after TCF7L2 knockdown, demonstrating that 1) the de novo prediction of TF-associated chromatin states (TF-states) by T-cep is valid; and 2) TCF7L2 are involved in regulating these genes enhancers (Fig. 5a). Out of the most significantly decreased expressed genes, we further chose KRT8, PTK2, ERRFI1 and ZNF217 for enhancer activity validations. We cloned specific TCF7L2-associated enhancer loci (TFE) into pGL3 promoter vector for enhancer efficiency detection and the cloned regions are listed in Additional file 1: Table S8-S9. We applied Luciferase Reporter Assay to further compare the activity between different types of TF-enhancers. In control cells, both cell types clearly illustrate TF-Intragenic enhancer shows more activity than TF-distal enhancers and ZNF217 TF-intragenic enhancer shows almost 4 times than ZNF217 TF-distal enhancer (Fig. 5b). Moreover, we applied TCF7L2 knockdown and TCF7L2 overexpression in both cell lines to characterize TCF7L2-associated enhancers. In TCF7L2 knockdown cell lines, we firstly wanted to confirm if TCF7L2 regulates the predicted intragenic and distal enhancers. The activities of all enhancers are decreased after TCF7L2 knockdown, notably the enhancers in PANC1 cells decreased more than those in MCF7 cells and both of ZNF217 and ERRFI1 intragenic enhancer decrease more than 70% in PANC1 cells, illustrating TCF7L2 indeed regulates those enhancer activities (Fig. 5b). In the other aspects, in TCF7L2 overexpression cells, clearly, both types of enhancers for four tested genes showed higher activities with TCF7L2 co-transfection. Strikingly, we found that TCF7L2 intragenic enhancers showed much higher activity than the TCF7L2 distal enhancers. The activity of KRT8 and PTK2 TF-intragenic enhancer increased more than 400% comparing their TF-distal enhancer around 300% in MCF7 cells. In PANC1 cells, ZNF217 and ERRFI1 intragenic enhancer also increase 30 ~ 50% although they are already on high basic activity level before TCF7L2 overexpressed (Fig. 5c). These exprimental results strongly supported a notion that TF intragenic enhancers might contribute higher in transcribing gene expression than TF distal enhancers. Taken together, our experimental validations not only confirmed the functionalities of annotated TCF7L2-associated enhancer states but also strengthened the reliability of our method in unveiling novel TF-states.

## Discussion

In this study, we have developed a novel computational method, T-cep, to identify transcriptional factor associated combinatorial epigenetic patterns from ChIP-seq data. T-cep applied an univariate HMM training, selected best models by the BIC score and imposed second HMM re-training for determine TF-state and non-TF-state. The unique features of T-cep are the following: (1) encoding each mark as an alphabet of 2^n^ observations as an input with ability of scaling up, (2) training HMM twice to reduce background noise and infrequent states, (3) outputting all combinatorial patterns for emission probability, and (4) marginalizing on decoding each mark probability in each state. This is a computationally intensive approach, allowing states in T-cep to directly examine combinatorial marks together, since they have emission probabilities for marks’ combinations rather than individual marks. This is in contrast to other software tools which are limited to treat all kind of data set as equal biological weight and lack a focus on the key factor during HMM training. Importantly, using the T-cep method, we were able to unveil a novel set of TF-associated intragenic enhancers that are predicted to be higher transcriptional activity than typical distal enhancers.

As a study case, TCF7L2 is chosen in that it is a key WNT downstream regulator which is implicated in cancers and diabetes, as well as its alternative splicing generates protein variants with differential promoter-binding and transcriptional activation [31, 32, 46]. After trained by T-cep with the TCF7L2-omics data, we uncovered four types of TCF7L2-associated chromatin states with distinct combinatorial characteristics and genomic distribution. Of them, TCF7L2 intragenic enhancer is particularly interesting. The Gene Enrichment and Pathway analyses further revealed that genes with TCF7L2-associated states are cancer type specific (Fig. 3a) and their intragenic enhancers are more associated with advanced stage markers in cancer such as Thyroid hormone signaling and Proteoglycan in cell adhesion (Fig. 3c), illustrating their functional relevance to specific cancer progression and metastasis. Our functional validations found that such intragenic enhancers exert higher transcriptional activities than those distal enhancers in TCF7L2 regulated gene expression. Our findings provide a new set of intragenic enhancers consisting of epigenetic signatures H3K4me1, H3K27ac and H3K36me3 [47] that may play more important functional role in diseases progression. This is in contrast to the role of conventional distal enhancers with typical epigenetic marks of H3K4me1, H3K27ac but the absence of significant H3K4me3 [48, 49] in regulating the cell type specificity.

One notable advantage of T-cep is that it can identify more TF-states at a cell-type dependent manner in comparison to ChromHMM due to the unique underlying algorithmic design. At a broader application, our T-cep was able to identify five MYC-associated states, states 1, 2, 4, 5 and 6, the similar pattern found in TCF7L2 data sets: TF-promoter (state 2), TF-intragenic enhancer (state 6), TF-distal enhancer (state 5) and TF-non-combinatorial state (state 1), as well as TF-inactive enhancer state (state 4) (Additional file 1: Figure S10). Three of these states (state 1, 4, 6) were noticeably missed by ChromHMM (Additional file 1: Figure S11). In a broader aspect, T-cep may be capable of deeply exploiting transcription factor associated epigenetic patterns where many other tools are unable to, and may further uncover their novel functionalities associated with various diseases or cancer types, providing a rationale to explore the underlying mechanisms.

## Conclusions

In summary, our study for the first time at a genome-wide scale reveals the enhanced transcriptional activity of cell type-specific TF-associated intragenic enhancers, allowing us more insights into underlying epigenetic regulatory codes in different cell or tissue types as well as in normal or disease conditions.

## Methods

### Training a hidden Markov model (HMM)

In HMMs, TF-state or non-TF-state are considered as hidden states and all of the possible combinations of chromatin marks are as observations. T-cep introduces a binary value for each segment that ‘1’ for mark presence or ‘0’ for mark absence based on a Poisson background distribution(*P* < 10^−4^). Observation symbols are made up by enumerating each possible combination of mark. For example, a segment with TCF7L2, H3K4me1 and H3K36me3 can be translated into 0b101000100, while 0 inside means no correspondent mark in this segment. The HMMs sequence is assumed as a segment-homogeneous first-order chain.

Let *n* be the number of distinct states and *m* be the number of distinct observation symbols per state in the model. We denote the individual hidden states as *S* = {*S*_*1*_,*S*_*2*_,…,*S*_*n*_} and individual combination histone mark symbols as *O* = {O_1_,*O*_*2*_,…,*O*_*m*_}. The hidden state transition probability matrix is *A* = {a_ij_}, where *A*_*ij*_ = *P*(*Y*_*t+1*_ = *S*_*j*_ | *Y*_*t*_ = *S*_*i*_), i ≥ 1, j ≤ n. The emission is the combination of TF, remodelers, histone marks symbol probability distribution. We assume *B* = {*b*_*j*_(*O*_*t*_)} is the symbol in hidden state S_j_, where *B*_*j*_(*O*_*k*_) = *P*(*X*_*t*_ = *O*_*k*_ | *Y*_*t*_ = *S*_*j*_), 1 ≤ j ≤ n, 1 ≤ k ≤ m. *X*_*t*_ is the Observation of symbol and *Y*_*t*_ represents the hidden state in bin *t*. To begin modeling, *π* is randomly initialized state probability distribution and there are three probability measures noted as *λ* = (*A*,*B*,*π*). Each HMM is trained for 300 iterations using a probability-scaling variant of the canonical Baum-Welch expectation-maximization algorithm with a minimum probability of 10^−6^ enforced for all transition, emission and initial probabilities to avoid potential numerical underflow. The best HMM is selected by the lowest Bayesian Information Criterion (BIC) score. After Viterbi decoding algorithm, those states with much less bins are removed from the model. Another canonical Baum-Welch algorithm is further modified by running multiple instances of the forward-backward algorithm. Let *ξ*_*t(i)*_ is the probability of the partial observation symbols sequence (*O*_*1*_,*O*_*2*_,…,*O*_*t*_) until bin *t* and the state *S*_*i*_ at bin *t*, given the model *λ. ξ*_*t(i)*_ can be written as *P*(*X*_*1*_ *= O*_*1*_ *, X*_*2*_ *= O*_*2*_ *,…,X*_*t*_ *= O*_*t*_ *, Y*_*t*_ *= S*_*i*_ | *λ*), the forward procedure can be calculated recursively by Eq 1, 2.

1 $$ {\xi}_{1(i)}={\pi}_i{b}_{i\ }\left({O}_1\right) $$

2 $$ {\xi}_{t+1(j)}={b}_j\left({O}_{t+1}\right){\displaystyle {\sum}_{i=1}^n{\xi}_{t(i)}{a}_{i j}} $$

We assume that the particular state (*Y*_*t*_ *= S*_*i*_) is the initialization. Let *ζ*_*t(i)*_ = *P(X*_*t+1*_ = *O*_*t+1*_,*X*_*t+2*_ = *O*_*t+2*_,…,*X*_*T*_ *= O*_*T*_|*Y*_*t*_ = *S*_*i*_, *λ*), to be the probability of the partial observation symbols sequence from *t + 1* to end, given the state *S*_*i*_ at bin *t* and the model *λ.* The backward procedure can be computed by Eq 3, 4.

3 $$ {\zeta}_{T(i)}=1 $$

4 $$ {\zeta}_{t(i)}={\displaystyle {\sum}_{j=1}^n{b}_j\left({O}_{t+1}\right){\zeta}_{t+1(j)}{a}_{ij}} $$

In order to re-estimate the HMMs parameter *λ*, the HMMs is trained for a further 100 iteration to produce the final states. We define *P*_(*Si,t*)_ as the probability of being in state *S*_*i*_ at bin t, given the model and the observation sequence and *P*_(*Sij,t*)_ is the probability of being in state *S*_*i*_ at bin t and state *S*_*j*_ at bin t + 1, given the model and the observation symbols sequence. We calculate the *P*_**(*Si,t*)**_ and *P*_**(*Sij,t*)**_ with the forward and backward variables *ξ*_***t(i)***_ and *ζ*_***t(i)***_. The HMM parameter *λ* can be re-estimated as following Eq 5:

5 $$ \left\{\begin{array}{l}\overline{\pi}={P}_{\left({S}_i, t\right)}\kern1.92em \left( t=1\right)\\ {}\overline{A}={\displaystyle {\sum}_{t=1}^{T-1}{P}_{\left({S}_{i j, t}\right)}}/{\displaystyle {\sum}_{t=1}^{T-1}{P}_{\left({S}_i, t\right)}}\\ {}\overline{B}={\displaystyle {\sum}_{t=1}^T{P}_{\left({S}_{i, t}\right)}}/{\displaystyle {\sum}_{\begin{array}{l} t=1\\ {}{X}_t= O\end{array}}^T{P}_{\left({S}_{i, t}\right)}}\end{array}\right. $$

The second Viterbi decoding algorithm is used to compute the highest probability of combinatorial epigenetic marks in correlation with the hidden states. Each combination of epigenetic mark has one output which is done to allow more direct interrogation of combinatorial epigenetic patterns with states. The probabilities of emitting each epigenetic mark independent are calculated by marginalization among all output combinations of marks probabilities. The independent emission probability follows Eq 6:

6 $$ {P}_{(ID)}={\displaystyle {\sum}_{x=1}^{2^n}\left\{\begin{array}{cc}\hfill {P}_{(x)}\hfill & \hfill \left(\left|{x}_{\&} ID>0\right.\right)\left.\right|\hfill \\ {}\hfill 0\hfill & \hfill \left({x}_{\&} ID=0\right)\hfill \end{array}\right.} $$

where *ID* is the epigenetic mark binary value, *x* is the output number and & is the bitwise *AND* operator.

### Implementation and Application

T-cep is implemented in C++, runs on Linux, and depends on the OpenMP and Boost APIs. T-cep contains a suite of scripts and program implementing the preprocessing, HMM training and TF-State calling steps. A univariate hidden Markov model (HMM) is applied to uncode observed genome-wide epigenetic data into hidden functional chromatin states. Several other scripts are provided for various utility functions.

For the concern of scaling limitation, although it is not necessary in this study (only 512 output combinations), T-cep also provided several scripts for allowing large numbers of marks in application. *getOutputMap.pl* can output the actually combination in all database. *manyOutputMapper.pl* used for mapping output combinations in precompiled datasets to an arbitrary ID system, which only for at least one bin actually found in output combinations. Due to far fewer mark combinations are expected to occur in practice, this script effectively frees T-cep from the scalability for reasonable outputs instead of considering every combination of marks as a possible output, which grows according to 2^n^. *ManyOutputModelBackConvert.pl* applied in mapping the outputs of HMMs trained with this data back to the original output combinations. This only considers mark combinations actually found in the data sets an upper bound to model complexity corresponding to the number of bins in all datasets.

The tool and scripts are available at our website, http://compbio.uthscsa.edu/T-cep/.

### Raw data processing

Public ChIP-seq datasets were downloaded from ENCODE project and only the 5’-end of uniquely mapped reads are used for the further analysis. Alignment to human reference genome hg19 was performed with Bowtie. Default settings were used with an exception that the number of allowed alignments was restricted to 1 in order to obtain only unique mapped reads, and seven threads were used. RNA-seq FASTQ files were aligned to hg19 using Tophat2 and average gene expression of each state was calculated by first finding the highest expression for any splice variant of each gene in the RNA-seq results. The midpoint of each bin is calculated, and compared to the 5’ and 3’ ends of annotated RefSeq genes. The average gene expression reported as gene expression levels are weighted by the number of bins close to them. The percentage of bins within RepeatMasker regions was calculated for all states in all cell lines. Hg19 RepeatMasker data was downloaded from http://www.repeatmasker.org/genomes/hg19/RepeatMasker-rm405-db20140131/. The raw RepeatMasker regions were extracted and converted to BED format. The mergeBed program of the BEDTools package was used to merge adjacent RepeatMasker regions. The merged regions were filtered to exclude regions less than 500 bp length. Correlation between state bins and these filtered regions was performed by BEDTools’ intersectBed.

### Co-transfection and RT-qPCR

TCF7L2 siRNAs were purchased from Thermo Fisher Scientific Silencer® Select siRNAs. For transfection of siRNA oligo, MCF7 and PANC1 cells were seeded by 6 cell plate with Lipofectamine® RNAiMAX Transfection Reagent for 48 h. Total RNAs from cells were extracted using Quick-RNA™ MiniPrep kit (Zymo Research). Then cDNA was prepared using RevertAid H Minus First Strand cDNA Synthesis Kit (Thermo Fisher Scientific). Expression of mRNA analysis was performed with LightCycler® 480 SYBR Green I Masteron and LightCycler® 480 System Sequence Detection System (Roche Applied Science) using GAPDH for normalization.

### Luciferase reporter assay

Intragenic or distal TFEs were transient transfection into MCF7/PANC1 cells with Lipofectamine 2000 reagent with or without TCF7L2 co-transfection and β-galactosidase expression vector. Cells were harvested and luciferase activity was determined using the Luciferase Assay System (Promega) per the manufacturer’s protocol. The β-galactosidase activity was performed on lysates as a control. The experiments were performed in triplicate.

## Additional files

Additional file 1: Supplementary Figures and Tables. (DOCX 4445 kb)

Additional file 2: Gene List associated with TF-states. (XLSX 330 kb)
