# Supplementary material for: Transcription factor-associated combinatorial epigenetic pattern reveals higher transcriptional activity of TCF7L2-regulated intragenic enhancers
Source: BMC Genomics. 2017 May 12;18:375. doi: 10.1186/s12864-017-3764-9 (PMC5429574; doi:10.1186/s12864-017-3764-9)
Supplement: Supplementary file 1 — Supplementary Figures and Tables. (DOCX 4445 kb) [file 12864_2017_3764_MOESM1_ESM.docx]

**Transcription factor-associated combinatorial epigenetic pattern reveals higher transcriptional activity of TCF7L2-regulated intragenic enhancers**

Qi Liu, Russell Bonneville, Tianbao Li, Victor X Jin

**Figure S1:** The log-likelihood of the 18-state HMM derived from the 25-state HMM at each training of second 100 iterations.

**Figure S2:** The transition (**A**) and initial (**B**) derived from the 25-state preliminary HMM.

**A:** The transitions are from the states on the y-axis to the x-axis.


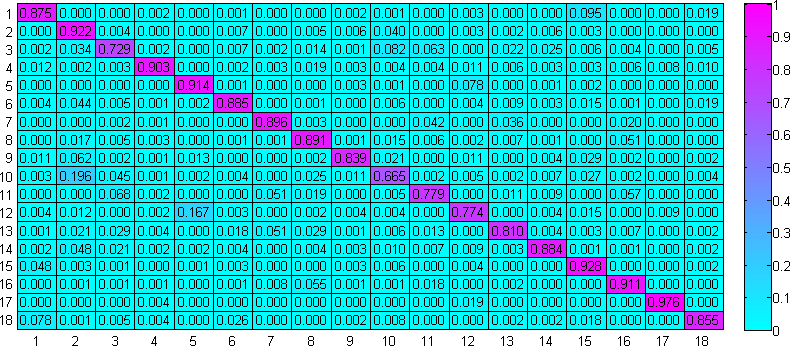


**B:** The initial probabilities of the 18-state HMM model.

| State: | 1 | 2 | 3 | 4 | 5 | 6 |
| --- | --- | --- | --- | --- | --- | --- |
| Prob: | 5.50E-06 | 4.44E-06 | 4.42E-06 | 4.46E-06 | 4.42E-06 | 6.09E-06 |
| 7 | 8 | 9 | 10 | 11 | 12 | 13 |
| 0.039225 | 4.42E-06 | 4.52E-06 | 4.42E-06 | 4.45E-06 | 4.44E-06 | 1.60E-05 |
| 14 | 15 | 16 | 17 | 18 |  |  |
| 0.042431 | 0.918258 | 4.94E-06 | 4.79E-06 | 9.05E-06 |  |  |

**Figure S3:** The emission probabilities of each output of the final 18-state HMM.


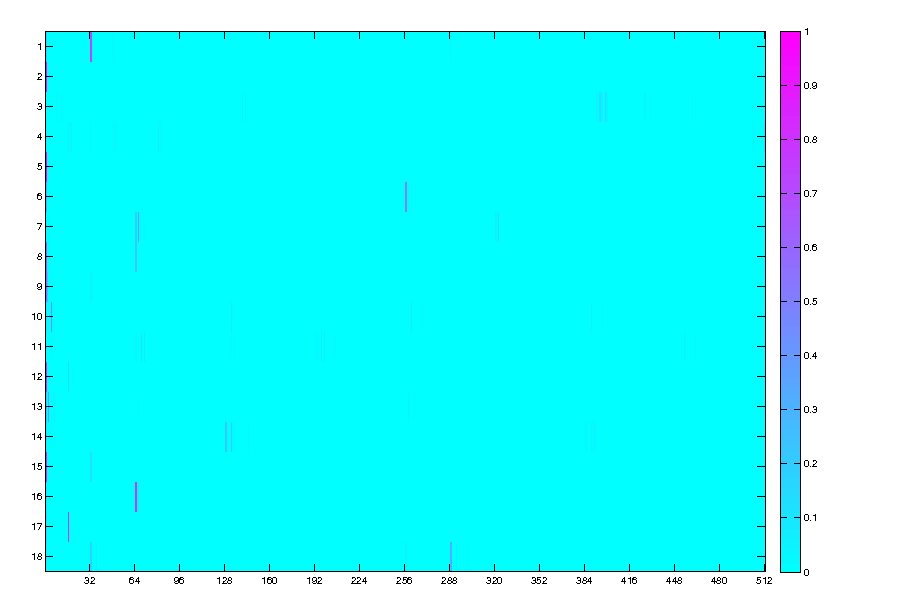


**Figure S4:** Plots of the output probabilities of each mark and the actual frequency of each mark in each cell line. The blue line is the expected line of best fit (perfect correlation, from (0, 0) to (1, 1)) and the red line is the actual line of best fit (calculated by least-squares regression).


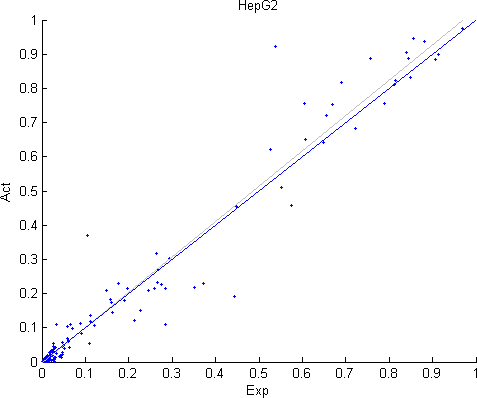


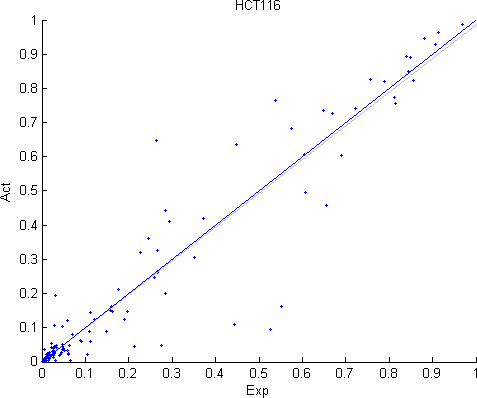


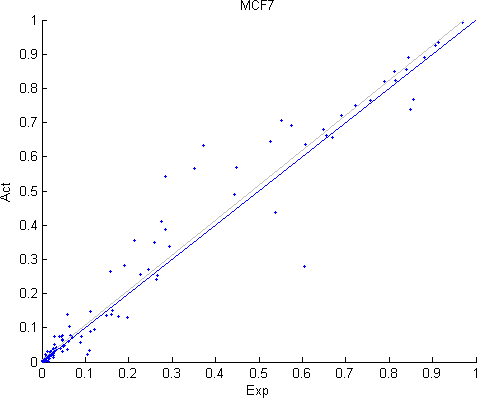


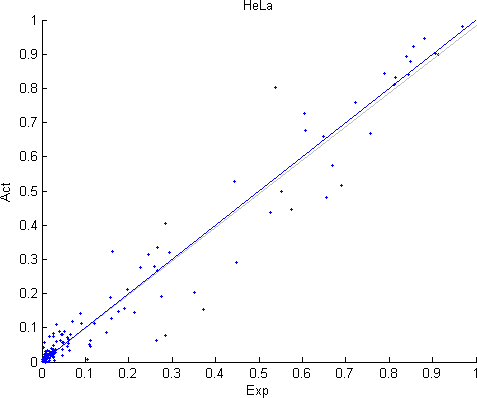


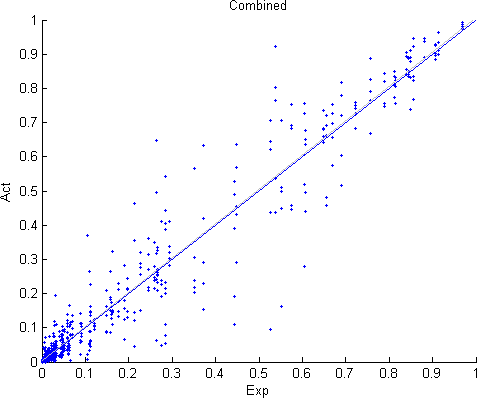

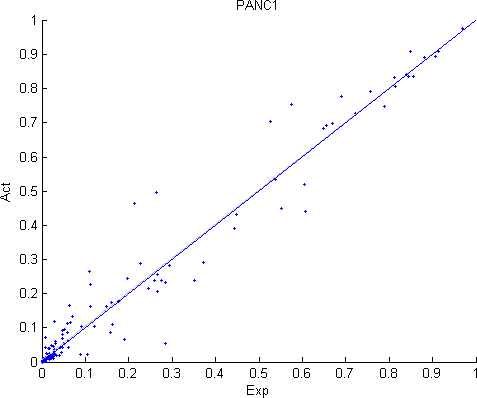


**Figure S5:** The mark frequencies in each cell line among bins in each state

**HCT116 HepG2**


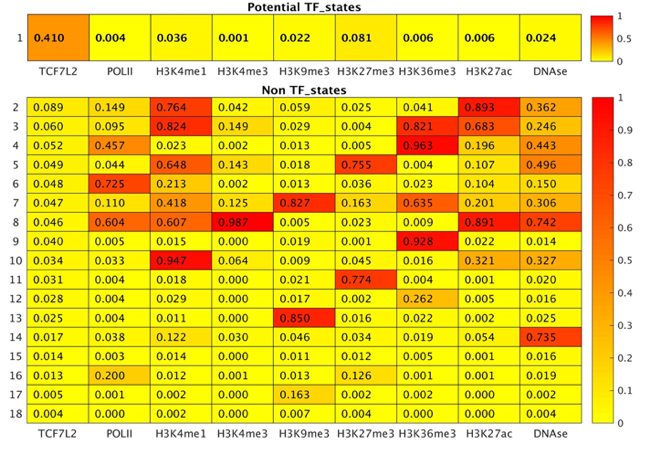

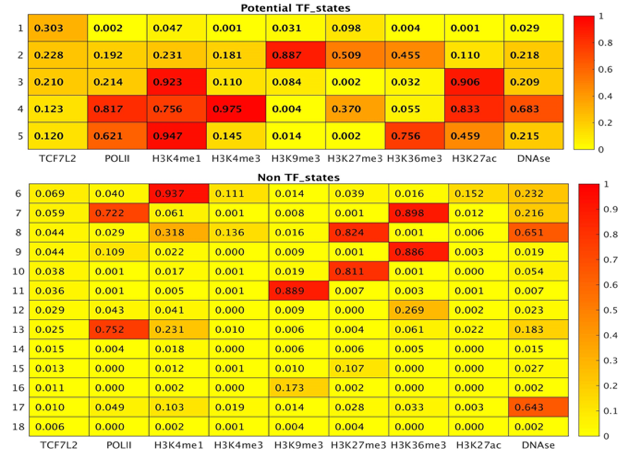


**HeLa MCF7**


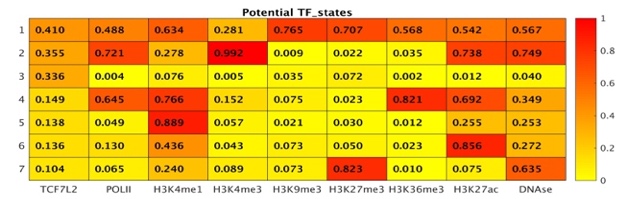

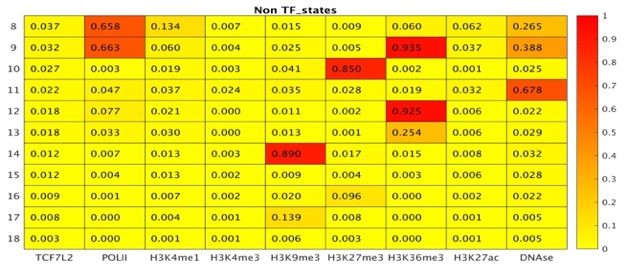

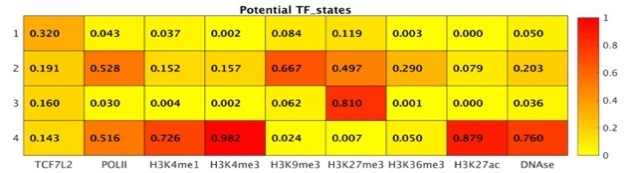

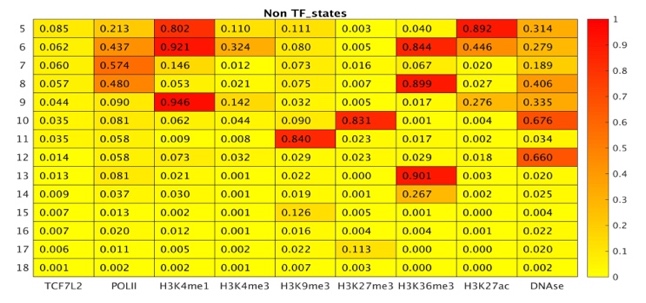


**PANC1**

**
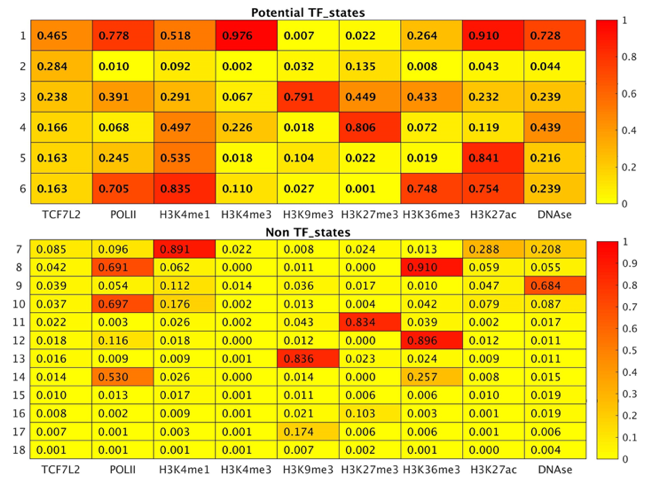
**

**Figure S6:** The correlations with other genomic information HMM states in all cells combined.


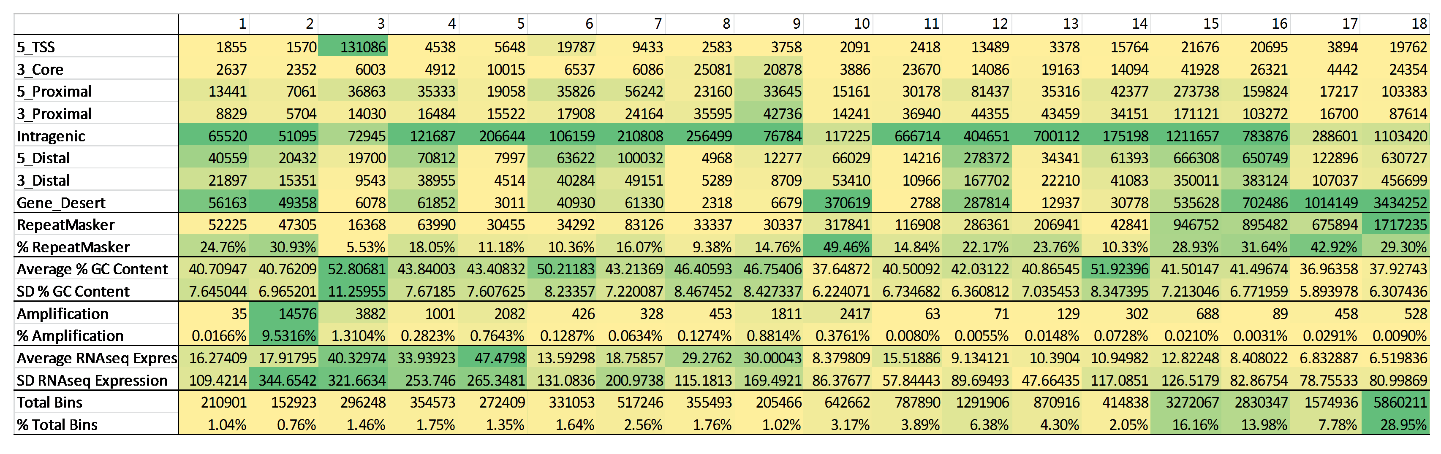


**Figure S7:** Plot of the distribution of states in all five cell types as described in Methods and Material. The body (5’ to 3’ with respect to the gene) and surrounding regions (+-90 kb) of each gene were divided into 120 bins spanning the region -90 kb to 5’ comprise the upstream surrounding region.

**
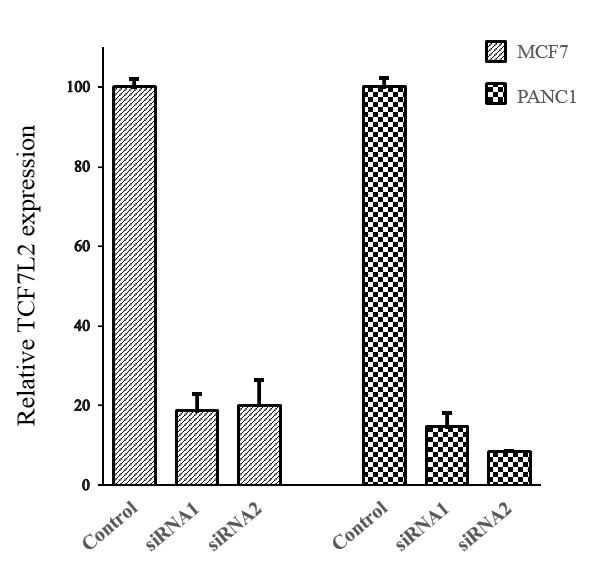
Figure S8:** TCF7L2 knockdown efficiency in MCF7 and PANC1 cell lines.

**Figure S9:** The training of TCF7L2-omics data by ChromHMM with initial 18-state model (left) and 25-state model (right), respectively.


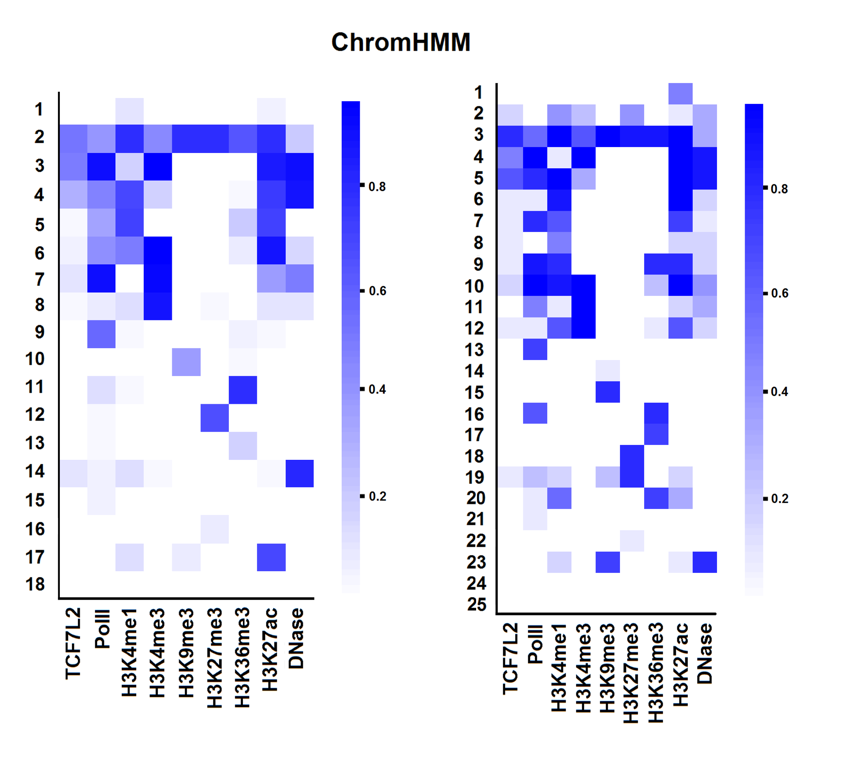


Some states can be assigned as a similar biological function in initial 25-state model. With an overall perspective, it showed that more ChromHMM states were annotated as undirected states in 25 states model. We did not find the clear difference of functional chromatin patterns between two models. It suggested an initial 18-state model is sufficient for ChromHMM in identifying the functional states.

**Figure S10:** For a broad test of T-cep, we applied T-cep on another data set, MYC-omics in four cell lines, HeLa, HepG2, K562 (not include in TCF7L2 data set) and MCF7.


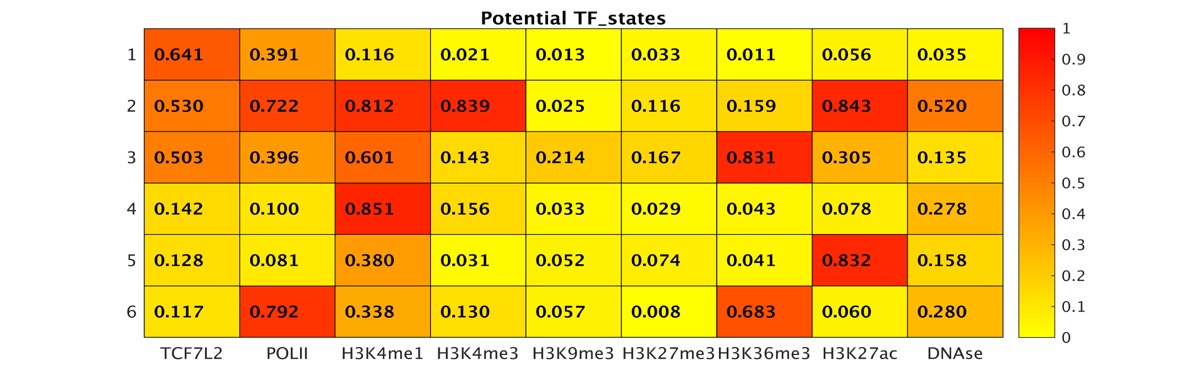

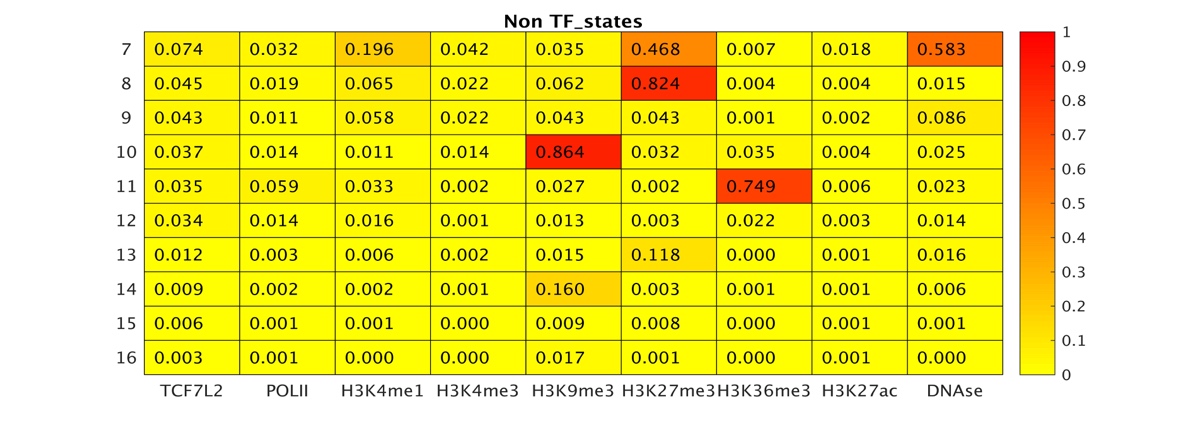


18-state HMM model was produced with BIC 3.68997*10^7^ after 300 iterations. With a cutoff of 0.1, we were able to classify five states associated with MYC, states 1-2, 4-6 as TF-states. For MYC-associated states, we can also identify the similar pattern as we did in TCF7L2 data set: TF-promoter (state 2), TF-intragenic enhancer (state 6), TF-distal enhancer (state 5) and TF-non-combinatorial state (state 1). One more TF-state found in MYC is state 4 as TF-inactivate enhancer.

State 2 was assigned as MYC-associated promoter because of its higher frequency in 5’-TSS regions and its high emission probabilities for PolII and H3K4me3. State 1 was classified as MYC binding, non-combinatorial TF-state, as its emission probability is only high for MYC and PolII. States 5 and 6 were classified as MYC-associated enhancer states due to their higher emission probabilities for the enhancer mark H3K27ac and H3K4me1. Location distribution showed most bins of state 6 were in gene body regions while the associated genes have a higher average gene expression and its high emission probabilities for enhancer marks H3K4me1, H3K27ac as well as gene body mark, H3K36me3. Thus, state 6 was classified as MYC-associated intragenic enhancer. State 5 was classified as a MYC-associated distal enhancer with H3K4me1 and H3K27ac. State 4 was assigned as inactivate enhancer due to absence of H3K27ac, which were not found in TCF7L2. State 3 was assigned as a mapping bias state.

**Figure S11:** A comparison with ChromHMM for MYC omics-seq data.


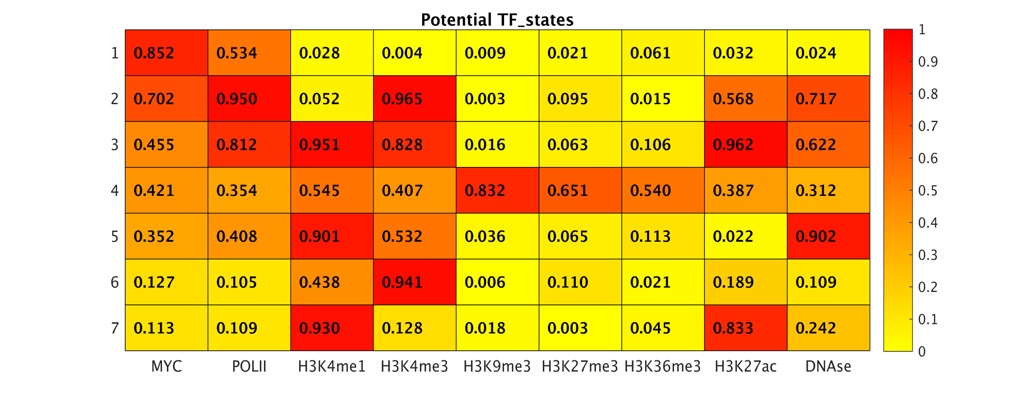

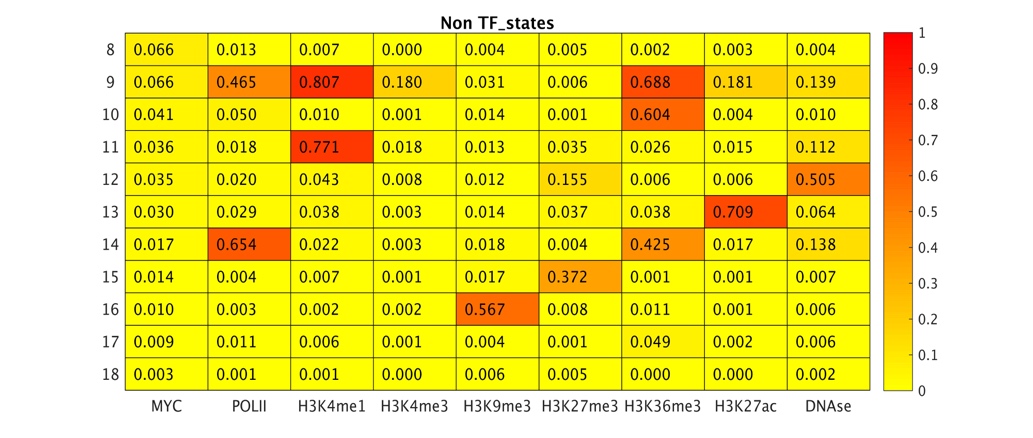


Although we can find seven TF-states (states 1, 2, 3, 4, 5, 6, 7) in MYC datasets, four (states 2, 3, 5, 6) of them are more focused on different TF-promoter states. In addition, state 9 with a high probability of H3K4me3, H3K36me3 and PolII was thus assigned intragenic enhancer. However, State 5 was not associated with MYC. In MYC dataset, T-cep also showed an advantage of predicting more TF-states including TF-intragenic enhancer. Taken together, the results of MYC and TCF7L2 showed the similar TF-state patterns, demonstrating a broader application of T-cep in successfully training different TFs in multiple cell lines. Therefore, T-cep can provide a robust tool for studying TF regulated combinatorial epigenetic regulation.

**Table S1**: Data sets were used in T-cep training.

|  | HeLa | HepG2 | MCF7 | HCT116 | PANC1 |
| --- | --- | --- | --- | --- | --- |
| TCF7L2 | GSM816436 | GSM782122 | GSM816438 | GSM782123 | GSM816437 |
| Pol-II | GSM733759 | GSM935603 | GSM822295 | GSM935426 | GSM1010788 |
| DNase | GSM736564 | GSM736639 | GSM1024767 | GSM736600 | GSM736519 |
| H3K4me1 | GSM798322 | GSM798321 | GSM946849 | GSM946853 | GSM818827 |
| H3K4me3 | GSM733682 | GSM733737 | GSM945269 | GSM945304 | GSM945856 |
| H3K27ac | GSM733684 | GSM733743 | GSM946850 | GSM946854 | GSM818826 |
| H3K27me3 | GSM733696 | GSM733754 | GSM946851 | GSM1323588 | ENCFF002AAI |
|  |  |  |  |  | ENCFF002AAJ |
| H3K9me3 | GSM1003480 | GSM1003519 | GSM946852 | ENCFF002AAK | ENCFF002AAG |
|  |  |  |  | ENCFF002AAM | ENCFF002AAH |
| H3K36me3 | GSM733711 | GSM733685 | GSM970217 | ENCFF002AAO | ENCFF002AAE |
|  |  |  |  | ENCFF002AAN | ENCFF002AAF |
| RNA-seq | GSM765402 | GSM758575 | GSM765388 | GSM958749 | GSM923421 |

**Table S2:** Number of unique mapped reads in each ChIP-seq experiment. Total reads: 2020399637.

|  | HCT116 | HepG2 | HeLa | MCF7 | PANC1 |
| --- | --- | --- | --- | --- | --- |
| TCF7L2 | 13045108 | 20840620 | 23283663 | 45532010 | 29674416 |
| Pol-II | 11327095 | 22126177 | 61911244 | 42889537 | 37457222 |
| H3K4me1 | 51343131 | 52320612 | 38435440 | 53456130 | 45010948 |
| H3K4me3 | 49150304 | 18620773 | 35897578 | 54134097 | 47338963 |
| H3K9me3 | 48699522 | 93907278 | 49032372 | 39213124 | 49944883 |
| H3K27me3 | 10526223 | 35259983 | 56820865 | 65774031 | 59766516 |
| H3K36me3 | 51066551 | 19355666 | 60030600 | 54120953 | 41487070 |
| H3K27ac | 35516872 | 14008928 | 39672909 | 59598739 | 54379908 |
| DNase | 80919605 | 50838536 | 54267867 | 89113893 | 53281675 |
| Total | 351594411 | 327278573 | 419352538 | 503832514 | 418341601 |

**Table S3:** BIC scores of all five preliminary 25-state HMMs. The highlighted HMM was selected due to its lowest BIC score.

| HMM: | s25_a | s25_b | s25_c | s25_d | s25_e |
| --- | --- | --- | --- | --- | --- |
| log likelihood: | 2.16E+07 | 2.19E+07 | 2.18E+07 | 2.15E+07 | 2.15E+07 |
| BIC: | 4.36E+07 | 4.40E+07 | 4.38E+07 | 4.32E+07 | 4.33E+07 |

**Table S4:** Number of bins in all cell lines in which each state in the preliminary 25-state HMM was called by the Viterbi algorithm. Highlighted states were manually removed, and transition probabilities to them uniformly redistributed to other states. This produced the initial 18-state HMM.

| State | 1 | 2 | 3 | 4 | 5 | 6 | 7 | 8 | 9 |
| --- | --- | --- | --- | --- | --- | --- | --- | --- | --- |
| Bins | 0 | 1307133 | 7195109 | 0 | 0 | 0 | 297068 | 156692 | 2921997 |
| State | 10 | 11 | 12 | 13 | 14 | 15 | 16 | 17 | 18 |
| Bins | 406525 | 0 | 330792 | 903017 | 188330 | 525948 | 279996 | 79 | 612185 |
| State | 19 | 20 | 21 | 22 | 23 | 24 | 25 |  |  |
| Bins | 222051 | 356377 | 3070989 | 827262 | 1 | 329340 | 311194 |  |  |

**Table S5:** Pearson correlation coefficients between the emission probabilities of the final 18-state HMM and the actual frequency of each mark in each cell line.

| Cell line | r^2^ |
| --- | --- |
| HCT116 | 0.9545 |
| HepG2 | 0.9784 |
| HeLa | 0.9755 |
| MCF7 | 0.9787 |
| PANC1 | 0.9795 |
| Combined | 0.9730 |

**Table S6**: The definition of genomic regions based on an annotated RefSeq gene.

| **Position Annotation** | **Relative distance annotated gene** |
| --- | --- |
| 5_TSS | 1000 (to 5’ end) |
| Intragenic | within gene |
| 5_Distal | 1000000 (to 5’ end) |
| 3_Distal | 1000000 (to 3’ end) |
| 3_Core | 2000 (to either end) |
| Gene_Desert | >1000000 |
| 5_Proximal | 10000 (to 5’ end) |
| 3_Proximal | 10000 (to 3’ end) |

**Table S7:** The primers for RT-qPCR.

| **Knockdown efficiency** |  |  |  |
| --- | --- | --- | --- |
| TCF7L2 forward | CATATGGTCCCACCACATCA |  |  |
| TCF7L2 reverse | CACTCTGGGACGATTCCTGT |  |  |
|  |  |  |  |
| **Selected loci in MCF7** | Primer F/R | **Selected loci in PANC1** | Primer F/R |
| CHD2 | F: CGAAAGCAGGCATTGGATCACT | CALM2 | F: TGCAGCATGGTTAGCTTTGT |
|  | R: GATGACAACTGTGTCCGCTGAA |  | R: TCAGTCAGTTGGTCAGCCTT |
| KRT8 | F: GATCTCTGAGATGAACCGGAACA | CD44 | F: TCCCTGCTACCACTTTGATG |
|  | R: GCTCGGCATCTGCAATGG |  | R: AACCAATCCCAGGTTTCTTG |
| MPRIP | F: CACACTCTGGCAGGAAGAAA | ERRFI1 | F: AAGACAGGCCTCCCAAAGTA |
|  | R: TTTGCTCTCTAGCCCAGGTT |  | R: TTGAGGTAAGACGGAAGGCT |
| PTK2 | F: GAAGGCCAATTTGGAGATGT | FHL2 | F: CCGTAGTGGAACGAAGGATT |
|  | R: CACGCTGTCCGAAGTACAGT |  | R: GGTCACTTCTCAGGAGGCAT |
| CSNK1D | F: CCGGTCTAGGATCGAAATGT | PARD6B | F: CGGCAAACCAGAGGAATAAT |
|  | R: AGCTCTTGGAGTCTGTCCGT |  | R: TTCAATCTGCTGTGGGTAGC |
| FAM102A | F: CAGAGCCAGGATTTCACAGA | SMAD3 | F: GTCCATCCTGCCTTTCACTC |
|  | R: TCACAACTGGCTCGTAGACC |  | R: CTTCTCGCACCATTTCTCCT |
| SNX27 | F: GAGCAGGCGAGAAGGAATTG | TNS3 | F: GGACCCACAAGACAGGAAGT |
|  | R: GCTTAGAACACAGCTGCCTC |  | R: GTCATGCTCTGCAAACAGGT |
| TMEM64 | F: TACCAATCTGTGGCTGTGGT | ZNF217 | F: CTACCCTCTGTGAACCAGCA |
|  | R: ATGACAATGCTTTGCACGAT |  | R: ATGTCTGCCCACATACCTCA |
| ZHX2 | F: AGCAGGTGGCTGACTTCTTT |  |  |
|  | R: GCCATTCACCTCTGTACCCT |  |  |

**Table S8:** Selected TF-enhancer loci for Luciferase experimental validation.

| **Seleted TF-enhancer loci** | **Genomic region** |
| --- | --- |
| KRT8 internal TFE | chr12:53295000,53295750 |
| KRT8 distal TFE | chr12:53314500,53315250 |
| PTK2 internal TFE | chr8:141744000,141744750 |
| PTK2 distal TFE | chr8:142051500,142052250 |
| ERRFI1 internal TFE | chr1:8073750,8075250 |
| ERRFI1 distal TFE | chr1:8180250,8181750 |
| ZNF217 internal TFE | chr20:52186500,52188000 |
| ZNF217 distal TFE | chr20:52237500,52239000 |

**Table S9**: The primer for TFE cloning.

| Gene loci | Primer sequence |
| --- | --- |
| KRT8_inter_F1 | GGAGACTCCCTCACCTTCTTG |
| KRT8_inter_R1 | ATCAACAACCTTAGGCGGCA |
| KRT8_dis_f1 | ACCGTCACATCCACAGCATT |
| KRT8_dis_r1 | GGGGTGGAGGTTTAGTGTGG |
| PTK2_inter_F1 | TGCCAGTCATGAAATAGCGT |
| PTK2_inter_R1 | TCTGTCAAGTACCAGCCACG |
| PTK2_dis_f1 | CGCTTCGTCCCTCTTAGTCC |
| PTK2_dis_r1 | ACCCTTTGAGGCATTTGCTTA |
| ERRFI1_inter_F1 | AGGTATGGGAACTCTGGGGG |
| ERRFI1_inter_R1 | GGCTGTGATTGCTCTTCGCT |
| ERRFI1_dis_f1 | GGACAAACTGCCACAAACCC |
| ERRFI1_dis_r1 | AAAGACCAGTGCTGTAGCCG |
| ZNF217_inter_F1 | TGTGAGCTGGTCCAGAGGAT |
| ZNF217_inter_R1 | CTGGATTGGCTGAGTGCGG |
| ZNF217_dis_f1 | CAGGAAGCCTCAAGGAGGAAG |
| ZNF217_dis_r1 | ATCCGGTTTGTAAGGGGCAA |
